# Structural Complications Following ST-Elevation Myocardial Infarction: An Analysis of the National Inpatient Sample 2016 to 2020

**DOI:** 10.3390/jcdd11020059

**Published:** 2024-02-15

**Authors:** Chun Shing Kwok, Adnan I. Qureshi, Maximillian Will, Konstantin Schwarz, Gregory Y. H. Lip, Josip A. Borovac

**Affiliations:** 1 Department of Cardiology, University Hospitals of North Midlands NHS Trust, Stoke-on-Trent ST4 6QG, UK; shingkwok@doctors.org.uk; 2 Zeenat Qureshi Stroke Institute and Department of Neurology, University of Missouri, Columbia, MO 65211, USA; qureshai@gmail.com; 3 Department of Internal Medicine 3, University Hospital St. Pölten, Karl Landsteiner University of Health Sciences, 3100 Krems, Austria; maximillian.will@me.com (M.W.); konstantin.schwarz@gmx.net (K.S.); 4 Karl Landsteiner Institute for Cardiometabolics, Karl Landsteiner Society, 3100 St. Poelten, Austria; 5 Liverpool Centre for Cardiovascular Science, University of Liverpool, Liverpool John Moores University & Liverpool Heart and Chest Hospital, Liverpool L69 3BX, UK; gregory.lip@liverpool.ac.uk; 6 Danish Center for Health Services Research, Department of Clinical Medicine, Aalborg University, 9220 Aalborg, Denmark; 7 Division of Interventional Cardiology, Cardiovascular Diseases Department, University Hospital of Split, 21000 Split, Croatia; 8 Department of Pathophysiology, University of Split School of Medicine, 21000 Split, Croatia

**Keywords:** ST-elevation myocardial infarction, complications, hemopericardium, ventricular septal rupture, cardiac wall rupture, mortality

## Abstract

ST-elevation myocardial infarction (STEMI) is a life-threatening emergency that can result in cardiac structural complications without timely revascularization. A retrospective study from the National Inpatient Sample included all patients with a diagnosis of STEMI between 2016 and 2020. Primary outcomes of interest were in-hospital mortality, length of stay (LoS), and healthcare costs for patients with and without structural complications. There were 994,300 hospital admissions included in the analysis (median age 64 years and 32.2% female). Structural complications occurred in 0.78% of patients. There was a three-fold increase in patients with cardiogenic shock (41.6% vs. 13.6%) and in-hospital mortality (30.6% vs. 10.7%) in the group with structural complications. The median LoS was longer (5 days vs. 3 days), and the median cost was significantly greater (USD 32,436 vs. USD 20,241) for patients with structural complications. After adjustments, in-hospital mortality was significantly greater for patients with structural complications (OR 1.99, 95% CI 1.73–2.30), and both LoS and costs were greater. There was a significant increase in mortality with ruptured cardiac wall (OR 9.16, 95% CI 5.91–14.20), hemopericardium (OR 3.20, 95% CI 1.91–5.35), and ventricular septal rupture (OR 2.57, 95% CI 1.98–3.35) compared with those with no complication. In conclusion, structural complications in STEMI patients are rare but potentially catastrophic events.

## 1. Introduction

ST-elevation myocardial infarction (STEMI) occurs when occlusion of a coronary vessel results in myocyte death, which may be life-threatening in the absence of timely coronary revascularization. There are between 44 and 142 cases of STEMI per 100,000 people depending on the European country [1], and in 2016, there were 163,715 patients with STEMI in the United States [2]. In-hospital mortality rates for patients with STEMI are estimated to be 5–6% in the United States [3] and 3–10% in Europe [4]. While advancements in pharmacology and catheter-based and surgical reperfusion have improved outcomes in acute myocardial infarction (AMI) over the decades, patients with large infarcts or those who do not receive timely revascularization still remain at risk of mechanical complications [5].

Mechanical complications of AMI include left ventricular free wall rupture, ventricular septal rupture, papillary muscle rupture, pseudoaneurysm, and true aneurysm [6]. Among these complications, three are life-threatening, which are ventricular free wall rupture, interventricular septum rupture, and acute mitral regurgitation [7]. One review suggests that they occur in fewer than 0.1% of patients and typically present with cardiogenic shock or acute pulmonary edema within the first week after myocardial infarction [6]. Data from the National Inpatient Sample (NIS) from the United States between 2003 and 2015 suggest that they occur in 0.27% of patients and have not significantly changed over time [8]. Since this study, there have been more contemporary data from the NIS available together with the conversion of ICD-9 to ICD-10 codes. The new codes under I23 describe certain current complications following acute myocardial infarction, which are superior compared with the ill-defined description and complications of heart disease used in the previous codes.

In this study, we conduct an analysis of nationally representative hospital data from the United States to evaluate any difference in in-hospital mortality, length of stay, and healthcare costs for adult patients who are non-electively admitted with STEMI according to the presence of structural complications. We also explored recent trends and comparative mortality rates for individual complications.

## 2. Materials and Methods

This manuscript is prepared in accordance with the recommendations of the STROBE criteria [9]. This work did not require institutional review board approval [10]. The NIS is a public dataset produced by the Healthcare Cost and Utilization Program in the United States, which contains more than 100 clinical and nonclinical variables, including diagnose and procedures, patient demographic characteristics, hospital characteristics, total charges, length of stay, and discharge status [11]. We analyzed the 2016 to 2020 NIS dataset for patients who were adults that were non-electively admitted to a hospital with any diagnosis of STEMI. We excluded patients whose records were missing age, sex, and death; those aged 18 years or younger; and admissions that were classified as elective.

The full description of the source of the included data is shown Table A1. We included hospital admissions with a STEMI based on ICD-10 diagnosis codes (I21.0, I21.1, I21.2, and I21.3). Structural complications were defined by the composite of codes I23.0, I23.1, I23.2, I23.3, I23.4, I23.5, and I23.6, which correspond to hemopericardium, atrial septal defect (ASD), ventricular septal rupture (VSR), cardiac wall rupture, ruptured chordae tendineae, ruptured papillary muscle, and intracardiac thrombus, respectively. Data obtained from the NIS dataset included age, sex, race, primary expected payer, income quartile based on ZIP code, hospital region, hospital bed size, rural hospital, and teaching hospital. ICD-10 diagnosis codes that were up to 40 were used to define comorbidities, including nicotine dependence, hypertension, hyperlipidemia, diabetes mellitus, atrial fibrillation, previous myocardial infarction, previous percutaneous coronary intervention (PCI), previous coronary artery bypass graft (CABG), heart failure, previous stroke, peripheral vascular disease, chronic lung disease, chronic kidney disease, malignancy, and dementia, and whether the patient was transferred from another hospital. ICD-10 procedure codes that were up to 25 were used to determine if the patient received thrombolysis or PCI. Other in-hospital events collected based on ICD-10 codes were cardiac arrest and cardiogenic shock.

The primary outcome of interest was in-hospital death, and the secondary outcomes were LoS and healthcare cost (defined by total charge multiplied by the charge-to-cost ratio).

### Statistical Analysis

Statistical analysis was carried out on Stata 13.0 (College Station, TX, USA). Discharge weights were applied to the dataset to obtain national estimates. The cohort was stratified by the presence or absence of structural complication. Descriptive statistics were presented according to these groups for demographic variables, hospital variables, comorbidity variables, and in-hospital events. Continuous variables were described with median and intraquartile range with the median test to determine if there were any significant differences among groups. For categorical variables, the percentage is presented, and the chi^2^ test was used to determine if there were any significant differences between groups. A *p*-value of <0.05 was considered as statistically significant. Trends between 2016 and 2020 were examined for the rate of structural complications overall and for specific complications. In-hospital mortality for structural complications was compared for 2016 and 2020. Multiple logistic regressions were used to determine the independent odds of mortality for structural complication compared with no complication. Multiple linear regression was used to evaluate the impact of structural complications on length of stay and cost. These analyses were adjusted for demographic variables, comorbidity variables, hospital variables, treatment variables, and in-hospital events. An additional multiple logistic regression was used to explore the independent differential impact of individual structural complications compared with no complication. We also defined the mean and standard deviation and the median and interquartile range for the length of stay according to different complications.

## 3. Results

**Figure 1:**
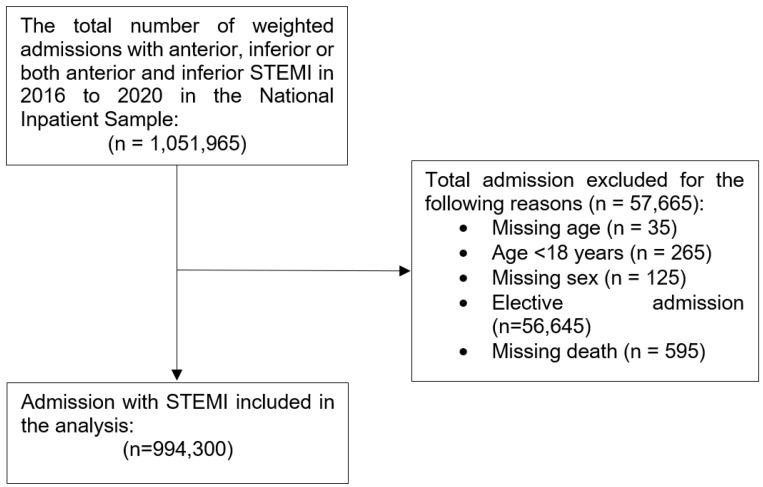
Flow diagram of hospital admissions with STEMI.

A total of 994,300 hospital admissions with STEMI between 2016 and 2020 were included in the final analysis (Figure 1).

The patient demographics, hospital characteristics, and comorbidities of the patients stratified by the presence or absence of structural complications are shown in Table 1.

Structural complications occurred in 0.78% for patients (n = 7805). The median age of the cohort was 64 years, and 32.2% were female. Patients with structural complications were greater in proportion in those admitted to large hospitals (68.0% vs. 54.1%, *p* < 0.001) and teaching hospitals (82.1% vs. 72.0%, *p* < 0.001). In terms of comorbidities, there was a greater proportion with atrial fibrillation (23.8% vs. 16.6%, *p* < 0.001), heart failure (56.0% vs. 28.8%, *p* < 0.001), and chronic kidney disease (17.3% vs. 15.4%, *p* = 0.038) in the group with structural complications but a lower proportion of hypertension (29.3% vs. 46.6%, *p* < 0.001), hyperlipidemia (51.6% vs. 62.6%, *p* < 0.001), nicotine dependence (23.4% vs. 29.3%, *p* < 0.001), and chronic lung disease (13.6% vs. 16.6%, *p* = 0.002).

The patient treatment and outcomes are provided in Table 2. There was nearly double (36.8% vs. 19.2%, *p* < 0.001) the proportion of patients who were transferred who had structural complications. In terms of treatment, there was no difference in the use of thrombolysis (1.0% vs. 1.2%, *p* = 0.49) but a lower proportion of patients with PCI (52.2% vs. 70.2%, *p* < 0.001) in patients with structural complications. While there was no statistical difference in cardiac arrest (8.5% vs. 7.2%, *p* = 0.055), there was a three-fold increase in patients with cardiogenic shock (41.6% vs. 13.6%, *p* < 0.001) in the group with structural complications, and in-hospital mortality was nearly three-fold greater (30.6% vs. 10.7%, *p* < 0.001). The median LoS was longer (5 days vs. 3 days, *p* < 0.001), and the median healthcare costs were significantly greater (USD 32,436 vs. USD 20,241, *p* < 0.001) for patients with structural complications.

After multivariable adjustments, in-hospital mortality was significantly greater for patients with structural complications (OR 1.99, 95% CI 1.73–2.30, *p* < 0.001), and both LoS and costs were greater (coefficient 2.05, 95% CI 1.73 to 2.37, *p* < 0.001; and coefficient 20.49, 95% CI 18.89 to 22.10, *p* < 0.001, respectively). For any structural complication, there was an increase in mortality from 27.7% in 2016 to 36.2% in 2020.

The trends in individual complications by year of admission are shown in Table 3. There was a slight increase from 0.13% to 0.20% in cases of VSR, an increase from 0.05% to 0.09% in ruptured cardiac wall, and a modest decline in patients with intracardiac thrombus from 0.46% to 0.40% when comparing 2016 and 2020. The proportion of patients receiving PCI increased from 65.9% in 2016 to 73.0% in 2018 and then decreased to 71.0% in 2020.

The rate of individual complications and the mortality rate associated with the complications are shown in Table 4. The structural complications ranged in frequency from 0.01% for atrial septal defect and ruptured chordae tendineae to 0.44% for intracardiac thrombus. The mortality rate with complications was the greatest for ruptured cardiac wall (65.5%), VSR (53.4%), and hemopericardium (51.1%).

**Figure 2:**
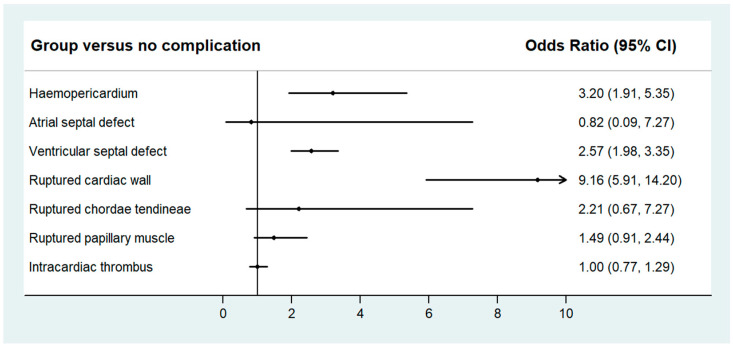
Multivariable-adjusted* odds of in-hospital mortality for individual complications compared with no structural complications in patients with STEMI. Multivariable model was adjusted for age, sex, race, weekend admission, season, primary expected payer, income quartile, hospital region, hospital bed size, rural hospital, teaching hospital, nicotine dependence, hypertension, hypercholesterolemia, diabetes mellitus, atrial fibrillation, previous myocardial infarction, previous PCI, previous CABG, heart failure, previous stroke, peripheral vascular disease, chronic lung disease, chronic kidney disease, malignancy, dementia, thrombolysis, PCI, cardiac arrest, cardiogenic shock, and hospital transfer.

Figure 2 shows the odds of in-hospital mortality compared with no structural complication. After multivariable adjustments, there was a significant increase in mortality with ruptured cardiac wall (OR 9.16, 95% CI 5.91–14.20, *p* < 0.001), hemopericardium (OR 3.20, 95% CI 1.91–5.35, *p* < 0.001), and VSR (OR 2.57, 95% CI 1.98–3.35, *p* < 0.001). There was a trend suggestive of increased mortality; however, it did not reach statistical significance in patients with ruptured chordae tendineae (OR 2.21, 95% CI 0.67–7.27, *p* = 0.19) or ruptured papillary muscle (OR 1.49, 95% CI 0.91–2.44, *p* = 0.11). There were no significant differences in mortality for atrial septal defect and intracardiac thrombus.

The length of stay according to different complications is shown in Table 5.

## 4. Discussion

Our large, nationwide, contemporary analysis of structural complications during hospitalization for STEMI provides several key findings. First, these complications are rare, occurring in less than 1% of patients, but they can be catastrophic events associated with high in-hospital mortality (30.6%). Second, structural complications significantly increase both length of hospital stay and healthcare cost. Third, patients with structural complications are more likely to be admitted to larger hospitals and teaching hospitals and are transferred from other hospitals. These patients also are more likely to have heart failure and atrial fibrillation and develop cardiogenic shock. Finally, in terms of trends, there was a modest rise in VSR and cardiac wall rupture in 2020, and among those patients with complications, the proportion of patients with in-hospital mortality increased from 27.7% in 2016 to 36.2% in 2020. These findings suggest that structural complications in STEMI are rare but are robustly associated with increased in-hospital mortality, prolonged length of stay, and higher financial costs of hospitalization.

Structural or mechanical complications in STEMI have been well described in the literature. An evaluation of post-infarction VSR that was treated with surgery or percutaneous repair in the United Kingdom from 16 centers described an in-hospital mortality rate of 48.1% with no difference in long-term mortality for patients treated with surgery or percutaneous repair [12]. It has been previously demonstrated that rates of mechanical complications following STEMI were significantly lower with primary PCI compared with thrombolysis [13,14]. The main limitation of this study is that what is not known is the proportion of all the patients who were not offered percutaneous or surgical management of their structural complication. There are other studies from the NIS that consider structural complications. Among 10,902 patients with STEMI and post-infarction VSD, the in-hospital mortality was 26.4%, which was significantly greater among patients with shock [15]. Papillary muscle rupture associated with acute myocardial infarction has been reported to have an incidence of 0.03% and was associated with increased in-hospital mortality, length of stay, and cost [16]. For intracardiac thrombus, a previous evaluation of the NIS data in a cohort that was not restricted to patients with STEMI reported that in-hospital mortality was 5.2%, and the mortality was higher for the subgroup of patients with a concomitant diagnosis of cerebral infarction (8.5%) [17]. Collectively considering the mechanical complications in 3,951,861 patients with STEMI, 0.27% experienced mechanical complications, which included papillary muscle rupture (0.05%), VSR (0.21%), and free wall rupture (0.01%), and there was no significant change over time in the overall incidence of mechanical complications [8]. In the current analysis, we found that overall structural complications occurred in 0.78% of patients, and when using the same definitions as the previous NIS study, there were 0.30% patients with mechanical complications.

A few notable observations can be made examining trends in structural complications. For any structural complications, there was year-on-year increase from 27.7% in-hospital mortality to 36.2% in 2020. A large analysis from the nationwide Spanish database showed similar dynamics as the trends in the rate of mechanical complications were increasing over the years, particularly in the STEMI setting, while in-hospital mortality for the group with structural complications reached nearly 60% [18]. It is not clear why more patients are dying in recent years, but this is an area that requires further research. There also appears to be modestly greater proportion of patients with VSD and ruptured cardiac wall in 2020. While the exact reason for this is uncertain, it may represent a delay to presentation for patients with STEMI because of the COVID-19 pandemic, which has been described by multiple studies [19,20,21].

The rate of PCI in STEMI and mortality presented is similar to that reported in a previous study [22]. In the previous study, for the year 2016, the rate of PCI in STEMI was 67.8%, and the rate of in-hospital mortality was 10.5%. In our current analysis of 2016 to 2020 data, we report the rate of PCI in STEMI to be 70.1%, and the rate of in-hospital mortality was 10.9%. It is notable that complications were associated with a high mortality rate of 30.5% compared with 10.7% with no complications. Length of stay is a complex issue because it can be affected by mortality. There are patients with no complications who have more severe coronary disease or ischemic damage secondary to STEMI; also there are patients who have more severe complications, and this would increase mortality. Early mortality would lower the length of stay.

The results from the current study have several clinical implications. Despite the strong evidence base to support emergency revascularization with primary PCI in STEMI, structural complications post-STEMI are not common but are important when consenting patients for procedures. The rare nature of these events is such that it is important to highlight to junior trainees or operators who do not perform many procedures to be informed how to manage them. The approach of using registries may be advantageous so that multiple centers can combine their data and share their experiences. From a local level practical perspective, most cardiologists should be aware of the availability of resources to handle complications, and pathways should be developed in sites to rapidly transport patients to surgical centers. There remains further work to understand whether complications that developed in patients could have been prevented or avoided, but this requires more granular data than are available in the NIS.

We are not aware of any recognized mechanism for ASD in myocardial infarction. One possible mechanism may be ischemia affecting the blood supply to the septum. It is possible that this is an incidental finding when echocardiography was performed in a patient who was admitted with STEMI. However, the nature of the ICD-10 codes is that I23.1 is defined by “Atrial septal defect as current complication following acute myocardial infarction.“ This code is likely to be reliable as most codes are used for billing purposes. Nevertheless there may be errors, and more studies are needed to better understand the potential mechanism and how the code is being used in real-world practices.

The presented findings from our study are generalizable to contemporary patients and practices in the United States. The United States has a unique multi-tiered healthcare system, ethnically diverse population, and geographic variation. The observations in the current study that the structural complications are rare events mean that a nationally representative dataset is necessary to capture sufficient events. The strength of the current approach is that the ICD-10 codes are that the category I23 specifically describes certain complications for myocardial infarction within the 28-day period, which suggests that those captured are likely to have reliable diagnoses. However, there may be underreporting of the true complication rate as not all sudden deaths following STEMI will be evaluated for these complications.

### Limitations

This study has several limitations. First, the observational design of the present study may be subject to potential confounding due to the presence of unmeasured factors that might have an impact on the outcomes and complications that were of our principal interest. However, we tried to overcome this issue by performing multivariable analysis that was adjusted for the comprehensive list of baseline variables. Furthermore, while we were able to capture the use of PCI and thrombolysis, we do not have any information about other medications or pharmacotherapy used during the index hospitalization that may have contributed to outcomes that we measured. Second, the NIS dataset does not provide information regarding the timing of the complication during hospitalization nor does it provide more granular information about the severity of STEMI or related structural complications. Third, some of the complications may represent natural progression of the partially treated or untreated STEMI, and others may be an iatrogenic complication secondary to primary PCI or other management. Fourth, there may be some patients who might be discharged prior to the identification of complication or who die in a hospital, and investigations do not take place to ascertain if a complication occurred. However, the ICD-10 codes used for the complications are more reliable than the previous ICD-9 codes as the older codes are less specific that there were certain complications attributed to the acute myocardial infarction. Nevertheless, it is possible that some of the complications such as ASD and VSD may have occurred independent of the myocardial infarction. Finally, we do not have detailed information regarding the reasoning for the management as decisions about ceiling of care or decisions about palliation may impact mortality.

## 5. Conclusions

Structural complications occur in less than one in a hundred patients who present with STEMI, and they are more common in patients presenting with heart failure and cardiogenic shock. In-hospital mortality, length of stay, and healthcare costs were significantly greater for patients with STEMI who developed structural complications compared with patients without structural complications. In more recent years, a greater proportion of patients with structural complications died in a hospital.

## Figures and Tables

**Table 1:** Patient demographics, hospital characteristics, and comorbidities of hospital admissions with STEMI stratified by the presence of structural complication.

Variable	Total Patient Cohort (*n* = 994,300)	No Structural Complication (*n* = 986,495)	Structural Complication (*n* = 7805)	*p*-Value
Median age (years)	64 [55 to 74]	64 [55 to 74]	65 [56 to 74]	0.025
Female sex	32.2%	32.2%	34.1%	0.111
Race White Black Hispanic Asian/Pacific Islander Native American Other	74.8% 9.6% 8.6% 3.0% 0.5% 3.5%	74.7% 9.6% 8.6% 3.0% 0.5% 3.5%	76.0% 8.5% 7.2% 3.2% 0.8% 4.3%	0.071
Weekend admission	28.2%	28.2%	24.6%	0.002
Season Spring Summer Fall Winter	25.1% 24.7% 24.5% 25.7%	25.1% 24.7% 24.5% 25.7%	26.8% 24.6% 23.1% 25.4%	0.398
Primary Expected Payer Medicare Medicaid Private insurance Self-pay No charge Other	47.8% 10.7% 31.4% 6.3% 0.5% 3.2%	47.7% 10.7% 31.5% 6.3% 0.5% 3.2%	49.3% 11.9% 29.3% 5.4% 0.2% 3.9%	0.027
ZIP Income Quartile 0–25th 26th–50th 51st–75th 75th to 100th	28.6% 27.3% 24.3% 19.8%	28.6% 27.3% 24.3% 19.8%	27.1% 26.9% 24.3% 21.7%	0.243
Hospital Region Northeast Midwest South West	17.4% 22.9% 39.7% 20.0%	17.4% 22.9% 39.7% 20.0%	23.5% 23.6% 32.2% 20.8%	<0.001
Hospital Bed Size Small Medium Large	16.2% 29.6% 54.2%	16.3% 29.6% 54.1%	9.8% 22.2% 68.0%	<0.001
Rural hospital	18.6%	18.6%	18.6%	0.950
Teaching hospital	72.1%	72.0%	82.1%	<0.001
Nicotine dependence	29.3%	29.3%	23.4%	<0.001
Hypertension	46.5%	46.6%	29.3%	<0.001
Hyperlipidemia	62.5%	62.6%	51.6%	<0.001
Diabetes mellitus	33.5%	33.5%	32.2%	0.281
Atrial fibrillation	16.6%	16.6%	23.8%	<0.001
Previous myocardial infarction	12.6%	12.6%	8.7%	<0.001
Previous PCI	15.0%	15.0%	15.5%	0.542
Previous CABG	4.8%	4.8%	2.4%	<0.001
Heart failure	29.0%	28.8%	56.0%	<0.001
Previous stroke	7.2%	7.2%	6.8%	0.503
Peripheral vascular disease	8.2%	8.2%	10.8%	<0.001
Chronic lung disease	16.5%	16.6%	13.6%	0.002
Chronic kidney disease	15.4%	15.4%	17.3%	0.038
Malignancy	7.4%	7.4%	6.7%	0.290
Dementia	4.4%	4.4%	3.5%	0.100

Abbreviations: PCI, percutaneous coronary intervention; CABG, coronary artery bypass graft.

**Table 2:** Patient treatment and in-hospital outcomes for hospital admissions with STEMI stratified by the presence of structural complications.

Variable	Total Patient Cohort (*n* = 994,300)	No Structural Complication (*n* = 986,495)	Structural Complication (*n* = 7805)	*p*-Value
Transfer	19.4%	19.2%	36.8%	<0.001
Thrombolysis	1.2%	1.2%	1.0%	0.493
PCI	70.1%	70.2%	52.2%	<0.001
Cardiac arrest	7.2%	7.2%	8.5%	0.055
Cardiogenic shock	13.8%	13.6%	41.6%	<0.001
In-hospital mortality	10.9%	10.7%	30.6%	<0.001
Median length of stay (days)	3 [2 to 5]	3 [2 to 5]	5 [2 to 11]	<0.001
Median cost (USD)	20,292 [14,183 to 30,945]	20,241 [14,166 to 30,777]	32,436 [19,244 to 65,754]	<0.001

Abbreviations: PCI, percutaneous coronary intervention; MAACE, major adverse cardiac and cerebrovascular events; USD, United States dollar.

**Table 3:** Rate of events by year for specific structural complications.

Variable	2016	2017	2018	2019	2020
Hemopericardium	0.03%	0.04%	0.05%	0.07%	0.03%
Atrial septal defect	0.01%	<0.01%	<0.01%	<0.01%	0.01%
Ventricular septal rupture	0.13%	0.15%	0.23%	0.20%	0.22%
Ruptured cardiac wall	0.05%	0.09%	0.06%	0.06%	0.09%
Ruptured chordae tendinae	0.01%	0.01%	0.02%	0.02%	0.01%
Ruptured papillary muscle	0.05%	0.05%	0.06%	0.04%	0.05%
Intracardiac thrombus	0.46%	0.45%	0.49%	0.38%	0.40%
Any complication	0.74%	0.78%	0.88%	0.75%	0.78%
Cumulative in-hospital mortality rate for any complication	27.7%	27.0%	32.2%	30.1%	36.2%

**Table 4:** Rate and mortality for specific structural complications.

Variable	Rate (%)	Mortality Rate without Complication (%)	Mortality Rate with Complication (%)	*p*-Value
Hemopericardium	0.05%	10.7%	51.1%	<0.001
Atrial septal defect	0.01%	10.7%	41.7%	0.001
Ventricular septal rupture	0.19%	10.7%	53.4%	<0.001
Ruptured cardiac wall	0.07%	10.7%	65.5%	<0.001
Ruptured chordae tendinae	0.01%	10.7%	37.5%	<0.001
Ruptured papillary muscle	0.05%	10.7%	44.7%	<0.001
Intracardiac thrombus	0.44%	10.7%	12.8%	0.045

**Table 5:** Length of stay according to the individual structural complications.

Complication	Mean Length of Stay (±SD)	Median Length of Stay [IQR]
No complication	4.6 ± 6.6	3 [2 to 5]
Hemopericardium	7.9 ± 11.2	4 [1 to 8]
Atrial septal defect	13.8 ± 19.9	5 [2 to 19]
Ruptured left ventricle	5.0 ± 7.8	2 [1 to 5]
Ventricular septal rupture	10.8 ± 14.0	6 [1 to 14]
Rupture chordae	12.5 ± 12.0	8 [3 to 19]
Rupture papillary muscle	10.8 ± 13.0	7 [2 to 14]
Thrombus	8.2 ± 8.6	5 [3 to 10]
Any complication	8.8 ± 10.8	5 [2 to 11]

## Data Availability

The data used in this analysis may be purchased from the Healthcare Cost and Utilization Project (HCUP) website. The authors do not have permission to share the data used for the analysis.

## Appendix A

**Table A1:** Codes for data analysis and their source.

Variable	Source	ICD-10 Code
ST-elevation myocardial infarction	I10_DX1/40	I21.0, I21.1, I21.2, I21.3
Structural complication	I10_DX1/40	I23.0, I23.1, I23.2, I23.3, I23.4, I23.5, I23.6
Hemopericardium	I10_DX1/40	I23.0
Atrial septal defect	I10_DX1/40	I23.1
Ventricular septal rupture	I10_DX1/40	I23.2
Raptured cardiac wall	I10_DX1/40	I23.3
Ruptured chordae tendinae	I10_DX1/40	I23.4
Ruptured papillary muscle	I10_DX1/40	I23.5
Intracardiac thrombus	I10_DX1/40	I23.6
Age	NIS Core	“AGE” variable
Sex	NIS Core	“FEMALE” variable
Race	NIS Core	“RACE” variable
Weekend admission	NIS Core	“AWEEKEND” variable
Season	NIS Core	“AMONTH” variable where Spring is March to May, Summer is June to August, Fall is September to November, Winter is December to February
Primary expected payer	NIS Core	“PAY1” variable
ZIP income quartile	NIS Core	“ZIPINC_QRTL” variable
Hospital region	NIS Hospital	“HOSP_REGION” variable
Hospital bed size	NIS Hospital	“HOSP_BEDSIZE” variable
Rural hospital	NIS Hospital	“PL_NCHS” variable where rural hospitals were those which were micropolitan or not metropolitan or micropolitan
Teaching hospital	NIS Hospital	“HOSP_LOCTEACH” variable where teaching hospital was defined by urban teaching hospital
Nicotine dependence	I10_DX1/40	F17
Hypertension	I10_DX1/40	I10, 0100, O109, I16, I67.4
Hyperlipidemia	I10_DX1/40	E78
Diabetes mellitus	I10_DX1/40	E08, E09, E10, E11, E13
Atrial fibrillation or flutter	I10_DX1/40	I48
Previous myocardial infarction	I10_DX1/40	I25.2
Previous PCI	I10_DX1/40	Z95.5, Z98.61
Previous CABG	I10_DX1/40	Z95.1
Heart failure	I10_DX1/40	I09.81, I11.0, I50
Previous stroke	I10_DX1/40	I69, Z86.73
Peripheral vascular disease	I10_DX1/40	I70, I71, I72.0, I72.1, I72.2, I72.3, I72.4, I72.8, I72.9, I73.1, I73.8, I73.9, I74.2, I74.3, I74.4, I76, I77.1, I77.71, I77.72, I77.73, I77.74, I77.79, I79, K55.1, K55.8, K55.9, Z95.82
Chronic lung disease	I10_DX1/40	J40, J41, J42, J43, J44, J45, J46, J47
Chronic kidney disease	I10_DX1/40	N18
Malignancy	I10_DX1/40	Z85
Dementia	I10_DX1/40	F01, F02, F03, G30, G31
Transfer	NIS Core	“TRAN_IN” variable
Thrombolysis	I10_PR1/25	3E03317, 3E06317
Percutaneous coronary intervention	I10_PR1/25	02703D6, 02703DZ, 02704D6, 02704DZ, 02703E6, 02703EZ, 02704E6, 02704EZ, 02703F6, 02703FZ, 02704F6, 02704FZ, 02703G6, 02703GZ, 02704G6, 02704GZ, 02713D6, 02713DZ, 02714D6, 02714DZ, 02713E6, 02713EZ, 02714E6, 02714EZ, 02713F6, 02713FZ, 02714F6, 02714FZ, 02713G6, 02713GZ, 02714G6, 02714GZ, 02723D6, 02723DZ, 02724D6, 02724DZ, 02723E6, 02723EZ, 02724E6, 02724E6, 02724EZ, 02723F6, 02723FZ, 02724F6, 02724FZ, 02723G6, 02723GZ, 02724G6, 02724GZ, 02733D6, 02733DZ, 02734D6, 02734DZ, 02733E6, 02733EZ, 02734E6, 02734EZ, 02733F6, 02733FZ, 02733FZ, 02734F6, 02733G6, 02733GZ, 02734G6, 02734GZ, 0270346, 027034Z, 0270446, 027044Z, 0270356, 027035Z, 0270456, 027045Z, 0270366, 027036Z, 0270466, 027046Z, 0270376, 027037Z, 0270476, 027047Z, 0271346, 027134Z, 0271446, 027144Z, 0271356, 027135Z, 0271456, 027145Z, 0271366, 027136Z, 0270376, 0271466, 027146Z, 0271376, 027137Z, 0271476, 027147Z, 0272346, 027234Z, 0272446, 027244Z, 0272356, 027235Z, 0272456, 027245Z, 0272366, 027236Z, 027246Z, 0272376, 027237Z, 0272476, 027035Z, 027247Z, 0273346, 027334Z, 0273446, 027344Z, 0273356, 027335Z, 0273456, 027345Z, 0273366, 027336Z, 0273466, 027346Z, 0273376, 027337Z, 027045Z, 0273476, 027347Z, 02703ZZ, 02704ZZ, 02713ZZ, 02714ZZ, 02723ZZ, 02724ZZ, 02733ZZ, 02734ZZ
Cardiac arrest	I10_DX1/40	I46.2, I46.8, I46.9
Cardiogenic shock	I10_DX1/40	R57.0
In-hospital mortality	NIS Core	“DIED” variable
Length of stay	NIS Core	“LOS” variable
Cost	NIS Core	Charge-to-cost ratio x “TOTAHG” (total charge variable in NIS Core)
